# Therapeutic effect of adenosine on experimentally induced acute ulcerative colitis model in rats^1^

**DOI:** 10.1590/s0102-865020190120000004

**Published:** 2020-02-14

**Authors:** Gulcin Ercan, Gurkan Yigitturk, Oytun Erbas

**Affiliations:** I MD, Department of General Surgery, University of Health Science Bagcilar Training and Research Hospital, Istanbul, Turkey. Scientific, intellectual, conception and design of the study; technical procedures; analysis and interpretation of data; manuscript writing; critical revision, final approval.; II PhD, Department of Histology and Embryology, Faculty of Medicine of Mugla Sitki Kocaman University, Mugla, Turkey. Acquisition, analysis and interpretation of data; technical procedures; histopathological examinations; critical revision; final approval.; III MD, Department of Physiology, Faculty of Medicine of Demiroglu Bilim University, Istanbul, Turkey. Conception and design of the study, statistics analysis, manuscript preparation and writing, critical revision, final approval.

**Keywords:** Colitis, Ulcerative, NF-kappa B, Tumor Necrosis Factor-alpha, Rats

## Abstract

**Purpose:**

To examine the therapeutic effect of external adenosine on an acetic acid-induced acute ulcerative colitis model in rats.

**Methods:**

Thirty male mature rats were divided into three groups as control, acute colitis (AC) and AC+adenosine group (AC+AD). AC was induced by rectal administration of 4% acetic acid (AA). 5mg/kg/day adenosine was performed i.p for 4 weeks to AC+AD group. Rectum and colon were excised for microscopic and histopathological histopathologic evaluations, and immunohistochemical analysis of nuclear factor kappa B (NF-kB). Blood samples were collected for biochemical detection of TNF-α, Pentraxin-3 and malondialdehyde (MDA) levels.

**Results:**

AC group had generalized hyperemia and hemorrhage with increased macroscopic and histopathological scores compared with control (P <0.0001) while adenosine treatment decreased these scores significantly (P <0.001), with reduced distribution of disrupted epithelium, leukocyte infiltrates, and focal hemorrhage. AC group showed significantly increased immunoexpression of NF-kB in rectum, plasma and tissue levels of TNF-α, plasma Pentraxin-3 and MDA levels (P <0.0001) while adenosine reduced these levels (P < 0.05).

**Conclusion:**

Adenosine appears to promote healing of colon and rectum exposed to AA-induced AC, suggesting a boosting effect of adenosine on the intestinal immune system to cure ulcerative colitis.

## Introduction

Ulcerative colitis is a chronic inflammatory disease of the gastrointestinal tract. Its etiology involves the basic clinical symptoms of rectal bleeding, diarrhea, weight loss and abdominal pain, and other signs include anemia, fever, joint pain, damage to mucus membranes, renal calculi, and osteoporosis^1,2^. Although there are several environmental, genetic, and immunological factors which play essential roles in the pathogenesis of ulcerative colitis^2,3^, the underlying etiology and pathogenesis remains essentially unknown.

The medical treatment of ulcerative colitis generally starts with 5-aminosalicylic acid, followed by steroids and immunomodulators. In addition, calcineurin inhibitors (cyclosporine A and tacrolimus), infliximab or surgery may be considered to intensify the therapy^4^. Some alternative approaches have been recommended for ulcerative therapy, such as transdermal administration of nicotine, omega-3 fatty acids with anti-inflammatory properties, and biological substances, including tumor necrosis factor alpha (TNF-α), interleukin (IL)-2 receptor, and anti-IL-12 and IL-6 antibodies^5-7^, and even experimental treatments with the flavonoid compounds such as quercetin^8^ and hawthorn-derived products found in plant extracts^9^. Despite this great deal of attention in the therapeutic approaches to the disease during the past years, the pharmacological treatment is still unsatisfactory^3^.

The acetic-acid-induced ulcerative colitis in rats is one of the common animal models of inflammatory disease in the literature^7-9^. This experimental model resembles human ulcerative colitis in macroscopy, histopathology, inflammation in the colonic mucosa and the production of reactive oxygen species (ROS)^9^. The abnormal release of proinflammatory cytokines such as TNF-α, the lipid peroxidation, a known biomarker of oxidative stress, and the total thiol molecule capacity are important mediators found in the pathogenesis of experimental ulcerative colitis of rats^8,9^.

The nucleoside adenosine, present in all cell types, has modulatory effects on numerous inter- and intracellular signaling pathways. Its central role is being the main compound of the intracellular energy source, adenosine-5’-triphosphate (ATP), which is released into the extracellular milieu following tissue damage or platelet degranulation, and plays a key role in the regulation of several biological processes. The products of the sequential dephosphorylation of ATP (i.e. adenosine, inosine, and hypoxanthine) have shown to further modulate cellular function, including providing protection against oxidant-induced cellular injury and reducing inflammatory cell infiltration and lipid peroxidation in animal models of colitis^10,11^. Substantial accumulation of these metabolites also occurs in tissues and body fluids following conditions such as ischemia and inflammation. Adenosine in particular has gained attention of researchers as it is considered a “retaliatory metabolite” since its accumulation following a tissue injury results in vasodilation, correspondingly increased oxygen levels and nutrient supplies, thus decreasing the tissue damage^12^. Thus, adenosine has shown to have potent anti-inflammatory properties in different experimental models^12,13^; however, its therapeutic role has not been demonstrated in any acute ulcerative colitis model. The aim of this study was to examine the therapeutic effect of adenosine on an acetic acid-induced acute ulcerative colitis model in rats. We hypothesized that external administration of adenosine has an anti-inflammatory and amendatory effect on the colon which was damaged by acetic acid treatment, probably through modulating the tissue levels of nuclear factor kappa B (NF-kB) and TNF-α, and the plasma levels of lipid peroxidation and pentraxin-3 (PTX3).

## Methods

All animal studies were strictly conformed to the animal experiment guidelines of the Ethics Committee for Animal Care of Ege University. All procedures were performed according to the Animal Experimentation Ethics Committee under protocol number 2012-004.

In this study, 30 male Sprague Dawley albino mature rats weighing 200–220 g were used. Animals were fed ad libitum and housed in pairs in steel cages, having a temperature-controlled environment (22 ± 2°C) with 12-h light/dark cycles.

### Experimental protocol

Acute colitis was induced by rectal administration of 4% solution of acetic acid (AA) (Sigma Chemical Co.) in a volume of 1ml in 20 rats^14^. No drug was administered to the remaining 10 rats, assigned as the control group (n=10).

After ether anesthesia, a soft catheter was introduced into the anus up to 6 cm, through which AA was slowly administered. Before taking the catheter out, 1ml air was applied in order to spread AA completely in the colon. Care was taken to avoid any leakage from the anus. Then, 20 rats were randomly divided into 2 groups. Group 1 (n = 10; acute colitis and saline group) were given 1 ml/kg saline treatment. Group 2 (n = 10; acute colitis and adenosine group) were given 5mg/kg/day adenosine (ABFEN FARMA, Ankara, Turkey). All treatments were performed i.p for 4 weeks, after which the animals were euthanized and blood samples were collected by cardiac puncture for biochemical analysis. Rectum and colon were excised for the histopathological and biochemical examinations.

### Macroscopic evaluation

The abdomen was opened and the colon was exposed. The colon was excised from its closest proximity to the rectum up to level of splenic flexure. The colon was then longitudinally dissected along its mesenteric border. After washing the mucosa with saline solution, macroscopic scoring was done with a magnifying glass on the colon and rectum. This scoring system was as follows: 0, no damage; 1, patch type superficial hyperemia; 2, generalized patch type hyperemic regions with normal mucosa in between; 3, generalized hyperemia and hemorrhage^15^. After macroscopic evaluation, full-thickness tissue samples were obtained from the distal parts of colon close to the rectum.

### Histopathological evaluation

Formalin-fixed rectum sections in 4 μm thickness were stained with hematoxylin & eosin. All sections were photographed with Olympus C-5050 digital camera mounted on Olympus BX51 microscope.

Histopathological scores were assigned using the following criteria of MacPherson and Pfeiffer^7^: 0, intact epithelium, no leukocytes or hemorrhage: 1, <25% disrupted epithelium, focal leukocyte infiltrates, and focal hemorrhage; 2, 25% disrupted epithelium, focal leukocyte infiltrates, and focal hemorrhage; 3, 50% disrupted epithelium, widespread leukocytes, and hemorrhage; 4, >50% disrupted epithelium, extensive leukocyte infiltration, and hemorrhage.

### NF-kB immunoexpression

For immunohistochemistry, the rectum sections were incubated with H_2_O_2_ (10%) for 30 min to eliminate endogenous peroxidase activity and blocked with 10% normal goat serum (Invitrogen) for 1 hour at room temperature. Subsequently, sections were incubated by the primary antibody (NF-kB, Santa Cruz Biotechnology; 1/100) for 24 h at 4°C. Antibody detection was performed with the Histostain-Plus Bulk kit (Invitrogen) against rabbit IgG, and 3,3’ diaminobenzidine (DAB) was used to visualize the final product. All sections were washed in PBS and photographed with an Olympus C-5050 digital camera mounted on Olympus BX51 microscope. Brown cytoplasmic staining in intestinal gland and epithelial cell was scored positive for NF-kB. The number of NF-kB (+) cells was assessed by systematically scoring at least 100 intestinal glands and epithelial cells per field in 10 fields of tissue sections at a magnification of 100x. Positive control was not used but a negative control staining was performed by omitting the primary antibody from the procedure to demonstrate that the reaction visualized was due to the interaction of the epitope of the target molecule and the paratope of the antibody reagent.

### Biochemical detection of TNF-α levels in plasma

Plasma TNF-α level was measured using commercially available enzyme-linked immunosorbent assay (ELISA) kit (Biosciences). The plasma samples were diluted 1:2 and TNF-α was determined in duplicate according to the manufacturer’s guide. The detection range for TNF-*α* assay was *<*2 pg/ml.

### Biochemical detection of TNF-α levels in rectum

The frozen rectum tissues were homogenized with a glass homogenizer in 1 ml of buffer containing 1 mmol/L of PMSF, 1 mg/L of pepstatin A, 1 mg/L of aprotinin, and 1 mg/L of leupeptin in PBS solution (pH 7.2), and then were centrifuged at 12,000 rpm for 20 minutes at 4°C. The supernatant was then collected and the total protein was determined utilizing the Bradford method. The levels of TNF-*α* in the tissue supernatants were measured using an ELISA kit (eBioscience, Inc, San Diego, CA) speciﬁc for rat TNF-α in a step by step fashion consistent with the protocol of the kit. According to the speciﬁcations given by the manufacturer, the inter-assay and intra-assay coefﬁcients of variation for TNF-α were 7.9–8.2% and 6.1–6.5%, respectively. The minimum limit of TNF-α detected for this assay was 30 pg/ml. The cytokine contents in the rectum tissue were expressed as pg/mg tissue.

### Measurement of plasma lipid peroxidation

Lipid peroxidation was determined in plasma samples by measuring malondialdehyde (MDA) levels as thiobarbituric acid reactive substances (TBARS). Briefly, trichloroacetic acid and TBARS reagent were added to the plasma samples, then mixed and incubated at 100°C for 60 min. After cooling on ice, the samples were centrifuged at 3000 rpm for 20 min and the absorbance of the supernatant was read at 535 nm. MDA levels were expressed as nM and tetraethoxypropane was used for calibration.

### Biochemical detection of pentraxin-3 levels in plasma

Plasma pentraxin-3 (PTX3) levels were measured in each 100 μl sample by standard ELISA apparatus at 450 nm by using a PTX3 kit (Uscn Life Science Inc., Wuhan, China). PTX3 levels were determined in duplicate according to the manufacturer’s guide. The detection range for PTX3 assay was 0.078- 5 ng/ml.

### Statistical analysis

Data analyses were performed using SPSS version 15.0 for Windows. The normality of variables was tested by Kolmogorov-Smirnov test. The groups of parametric variables were compared by Student’s t test and analysis of variance (ANOVA). The groups of nonparametric variables were compared by Mann Whitney U test. Results were given as mean ± standard error of mean (SEM). A value of P < 0.05 was accepted as statistically significant. P < 0.001 was accepted as statistically highly significant.

## Results

All animals (n=30) in this 4 week-study survived at the end of procedures and there was no need to exclude none due to any inflammation or any alteration in feeding or weighing or complication of application by acetic acid or anesthesia protocol.

### Microscopic findings

**Figure 1:**
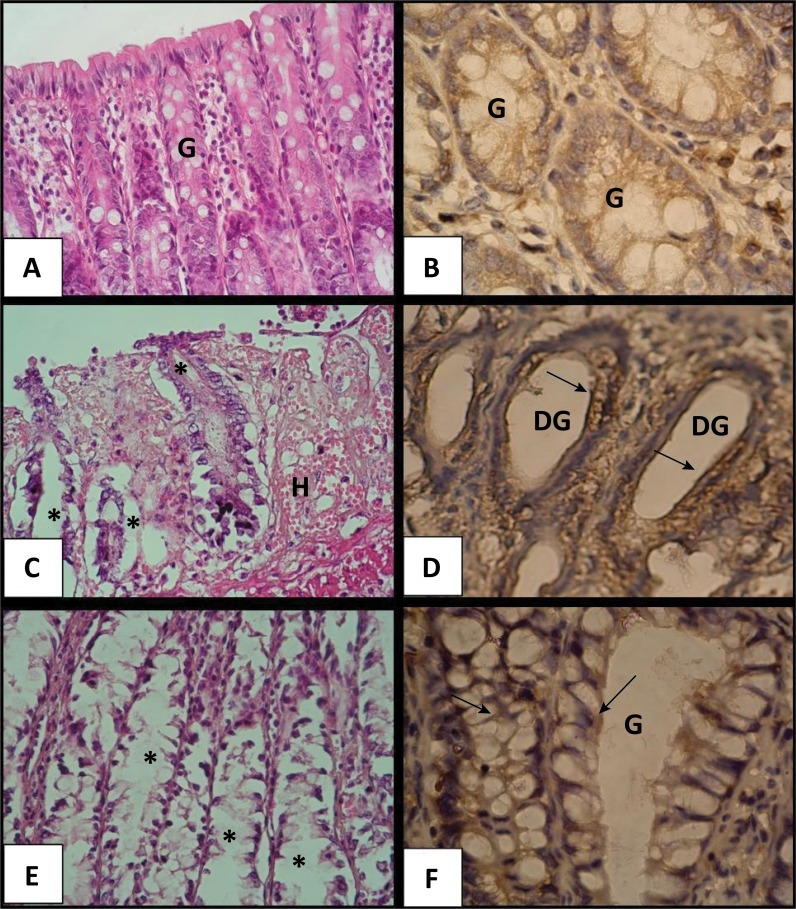
Microscopic images of rectum sections of rats with acute ulcerative colitis. a: The control group with the normal intestinal glands (G), b: Control group with low expression of NF-kB in the normal intestinal glands, c: Colitis and saline treatment group with damaged intestinal glands (*asterisks*) and severe hemorrhage (H), d: Colitis and saline treatment group with damaged intestinal glands (DG) and increased expression of NF-kB (*arrows*), e: Colitis and adenosine treatment group with healing intestinal glands (*asterisks*) and reduced hemorrhage, f: Colitis and adenosine treatment group with decreased expression of NF-kB (*arrows*) in healing intestinal glands (G). a, c, e: Hematoxylin and eosin stain (X40); b, d, f: NF-kappa B immunohistochemistry (X100).

Microscopic evaluation showed that the acute ulcerative colitis was successfully induced by 4% AA in 20 rats (Fig. 1). The scoring system of rectum and colon tissues also revealed that the acute colitis group treated with saline had generalized hyperemia and hemorrhage with an increased score compared with the control group (P < 0.0001) (Table 1). However, the treatment with adenosine decreased this score significantly compared to the colitis group treated with saline (P < 0.001); only patch type superficial hyperemia was observed in the intestinal normal mucosa.

**Table 1:** Macroscopic, histopathological and immunohistochemical scores of tissues.

	Control Group	Colitis and saline treatment group	Colitis and adenosine treatment group
Macroscopic score	0.18 ± 0.09	2.6 ± 0.4*	1.85 ± 0.54^##^
Histopathological score	0.2 ± 0.08	3.45 ± 0.3*	2.38 ± 0.65^#^
Immunohistochemistry NF-kB expression (%)	15.6 ± 3.8	78.6 ± 7.5*	24.5 ± 6.2^##^

*P < 0.0001, Compared with the control group.

#P < 0.05, ## P < 0.001, Compared with the colitis and saline group.

### Histopathological findings

Histopathology also showed that acute ulcerative colitis was successfully induced by AA (Fig. 1). The histopathological scoring system of rectum tissues revealed that the acute colitis group treated with saline had 50% disrupted epithelium, widespread leukocytes, and hemorrhage with an increased histopathological score compared with the control group (P < 0.0001) (Table 1). However, the treatment with adenosine decreased this score significantly compared to the colitis group treated with saline (P = 0.023). The ratio of distribution of disrupted epithelium was reduced up to 25% and there were focal leukocyte infiltrates, and focal hemorrhage in the adenosine treatment group (Fig. 1). There was no significant difference among the control and adenosine groups in terms of histopathological scores.

### NF-kB immunoexpression

Immunohistochemistry for NF-kB in the rectum showed that the colitis and saline treatment group had significantly increased immunoexpression of NF-kB in intestinal glands and epithelial cells (P < 0.0001) while the adenosine treatment dramatically reduced this expression compared with the colitis and saline group (P < 0.001). There was no significant difference among the control and adenosine groups in terms of immunohistochemical scores (Table 1).

### Biochemical findings

Table 2 represents all biochemical findings of the study. According to the results, plasma and rectum TNF-α levels increased significantly in the colitis and saline treatment group, compared with the control group (P < 0.0001 and < 0.001, respectively), while the adenosine treatment decreased these levels nearly down to the control levels (P = 0.018).

**Table 2:** Biochemical results for the plasma and tissues.

	Control Group	Colitis and saline treatment group	Colitis and adenosine treatment group
Plasma TNF-α (pg/ml)	22.8 ± 4.05	68.5.3 ± 8.02**	38.6 ± 7.1^#^
Rectum TNF-α (pg/mg tissue)	123.4 ± 11.3	195.5 ± 10.6*	158.1 ± 8.9^#^
Plasma MDA (nM)	67.1 ± 4.7	132.2 ± 5.8**	84.6 ± 8.1^##^
Plasma Pentraxin-3 (ng/ml)	1.5 ± 0.23	3.2 ± 0.24 *	2.1± 0.65 #

*P < 0.001, **P < 0.0001 Compared with the control group.

#P < 0.05, ## P < 0.001 Compared with the colitis and saline group.

Comparing the lipid peroxidation levels in plasma among groups (Table 2), MDA level in the colitis and saline treatment group rose significantly compared with the control group (P < 0.0001), while the adenosine treatment decreased this level nearly down to the control levels (P < 0.001). Plasma PTX3 levels also increased significantly in the colitis and saline group (P < 0.001) while decreased in the adenosine treated rats with colitis (P < 0.05).

## Discussion

Released by cells into the extracellular space during hypoxia, ischemia, or exercise, the nucleoside adenosine is a potent key regulatory molecule to balance the cellular oxygen supply and demand during the metabolic stress^11^. During the acute inflammation process, neutrophils release the nuclear DNA to trap the pathogens, providing a rich source for extracellular nucleotides and nucleosides. Nucleosides are also supplied luminally from dietary sources. Therefore, under a variety of pathological and nonpathological conditions, a large number of adenosine molecules may accumulate in the extracellular space in the vicinity of both the apical and basolateral surfaces of intestinal epithelial cells^12^. Since endogenously generated adenosine is suggested to play a role in the regulation of inflammation in the intestine, until now, the attempts to enhance the adenosine signaling during experimental colitis have exerted many beneficial results^13^. Aiming to support these results in this experimental study, we showed that the long-term adenosine treatment could ameliorate AA-induced colitis ulcerative pathologies in the colon and rectum of rats. The therapeutic effect of adenosine on experimentally induced colitis was reflected in its ability to decrease the AA-induced hyperemia and hemorrhage, disruption of intestinal epithelium, infiltration of leukocytes into colon and rectum, AA-increased NF-kB immunoexpression, and AA-elevated TNF-α in both plasma and rectum tissues, MDA and PTX3 levels in plasma.

The major histopathological changes observed in ulcerative colitis are the ulceration and erosion of the mucosa of mostly the rectum and the distal colon. Substantial dilation and thinning of the intestinal walls, inflammatory exudates, coarsened epithelium, hyperemia, edema, friable mucus membranes with hemorrhage are the other symptoms reported in the colons of rats with experimentally induced colitis^16^. According to our results, we also observed high scores in the microscopic analysis of colon and rectum, implicating that AA induced a generalized hyperemia and hemorrhage in the colon and rectum. High scores of histopathology also confirmed that AA impaired the integrity of intestinal epithelium, the immune system and the vascularization, hence, yielded a hemorrhage.

By far, AA-induced ulcerative colitis has been used eminently in many experimental studies^3,7,9,14,15^. However, the effects of adenosine treatment in this model were not reported before. Therefore, the current study is the first one to demonstrate these effects in AA induced ulcerative colitis in the colon of rats. Our results including microscopic and histopathological findings confirmed that acute ulcerative colitis was successfully induced in the animals.

The production of adenosine is considered to provide a potent anti-inflammatory and tissue-protective function in the mucosal inflammation of intestinal diseases^17^. Adenosine synthesis as well as adenosine receptor molecules involved in this inflammation process are responsible for the inflammation-dampening effects of adenosine^18^. The simultaneous action of adenosine in immune response suppression re-directs the adaptive T cell-mediated immune response, showing the strong interrelation between inflammation and tissue metabolism^19^. An approach for the treatment of intestinal inflammation diseases based on influencing the purinergic signaling pathways has gained attention^20^. The relation between the extracellular signaling mediated by nucleosides such as adenosine and the signaling pathways in the circulatory system may contribute to the immune response modulatory function of these nucleosides. Adenosine-dependent treatment, such as using agonists of adenosine receptor A2A, was reported to ameliorate the experimental colitis, while experimentally genetic alterations in adenosine signaling due to single nucleotide polymorphisms in intestinal inflammatory diseases was reported to increase the severity and susceptibility of disease^21^. In the present experimental study, adenosine treatment appears to promote the healing of the colon and rectum which was exposed to AA, compared with the non-treated ulcerative colitis group, suggesting that a boosting effect of adenosine on the intestinal immune system to cure the colitis.

In addition to their absorptive functions of the gastrointestinal tract, intestinal epithelial cells are also vital barriers for the mucosal immune system^22^. These cells express various adhesion molecules, receptors, the adenosine receptors and pro-inflammatory mediators allowing them to function for the immune system^23^. Extracellular adenosine interacts with immune cells via both specific G-protein coupled surface receptors^24^ and through the uptake of adenosine through nucleoside transporters^25^. There are several studies revealing that NF-kB is a critical mediator of the pathogenesis of intestinal inflammation, and therefore, it is suggested as an attractive therapeutic target for the treatment of various intestinal inflammatory disorders in humans^12^. Thus, we examined the immunoexpression of NF-kB in the rectum tissues of experimentally induced ulcerative colitis, and determined that the adenosine treatment attenuated AA-elevated immunoexpression of NF-kB.

As an irritant agent, AA induces the colitis involving infiltration of colonic mucosa with neutrophils and increased production of inflammatory mediators, such as hydrogen peroxide, nitric oxide (NO), myeloperoxidase, and TNF-α. In a study by Kuralay *et al*.^15^, trimetazidine, an antianginal compound, was administered to investigate its cytoprotective features in AA-induced colitis in rats, and found that AA administration markedly lowered TNF-α levels while trimetazidine treatment elevated these levels significantly. In another study by Southey *et al*.^26^, enhancement of TNF-α release was partially NO dependent in activated neutrophils in the chronic colitis and inflammatory bowel disease, hence it may be speculated that AA interferes with NO activity to reduce TNF-α levels. Moreover, extracellular adenosine/A2BR, the predominant adenosine receptor expressed on intestinal epithelial cells, also reduces intestinal epithelial cell expression of TNF-α, as well as its activity on macrophages dampens TNF-α levels. The activity of another adenosine receptor expressed by most immune cells, A2AR, on neutrophils inhibits neutrophil adhesion to endothelial/epithelial cells and destroys these cells, as well as modulates the production and release of TNF-α. Similarly, in our study AA administration markedly lowered TNF-α levels both in plasma and tissues; however, adenosine treatment restored the levels to control values^27^. Here, the amelioration of AA-induced colitis by adenosine might involve their receptor bindings or inhibition of NO but we did not measure the receptor or NO amounts in cells, and this needs to be investigated by further molecular studies.

There are several studies that have demonstrated the ability of adenosine to exert anti-inflammatory actions in a variety of animal models. These anti-inflammatory effects can be exerted by increasing intracellular or extracellular adenosine levels through the mechanism of either elevating the production of adenosine or inhibiting adenosine catabolism. Most of reports have been focusing on direct activation of adenosine receptors or inhibition of adenosine catabolism. The inhibition of adenosine deaminase was reported to attenuate mucosal inflammation in experimental colitis through the mechanism of reducing the production of MDA and TNF-α levels^28^. Considering the biochemical findings of the present study, it is worthwhile to conclude that the adenosine exerts its therapeutical effects partly by decreasing the MDA and PTX3 levels to reverse the detrimental effects of AA. Consistent with this finding, our histopathological findings confirmed that the adenosine-treated animals had less pathologic sign of inflammation, edema, hemorrhage and epithelial damages compared with non-treated animals with colitis. The exact mechanism of these effects is not clear but our results suggest adenosine may interfere with the lipid peroxidation in cells. Lipid peroxidation, a known biomarker of oxidative stress and indicated by MDA content of cells, is used in the evaluation of oxidant/antioxidant effects which are altered in the inflammatory intestinal diseases^9^. As we have shown here, the adenosine treatment attenuated the colitis-elevated MDA content in plasma.

Pentraxins (PTXs) are a family of highly conserved multimeric pattern recognition proteins, divided into two groups. The prototype of the long PTX family is PTX3, which is rapidly produced and released by diverse cell types at sites of inflammation, especially by endothelial cells, smooth muscle cells, mononuclear phagocytes, and dendritic cells in response to primary inflammatory signals such as TNF-α^29^. PTX 3 interacts with various ligands, including growth factors, extracellular matrix components and selected pathogens, resulting in complement activation, pathogen recognition by phagocytes and apoptotic cell clearance^29^. Savchenko *et al*.^30^ reported the expression of PTX3 mainly in neutrophils and less in macrophages recruited to colonic mucosa of patients with ulcerative colitis. In addition, they found that the numbers of PTX3-expressing cells and inflamed neutrophils were increased in the high histological grades of the inflammatory reaction, suggesting that PTX3 expression based on the colon tissue responds to inflammation. Moreover, most of neutrophils presented in the crypt abscess were positive for PTX3 antibody, suggesting that neutrophils are the main cellular source of PTX3 in human gastrointestinal tissue of colitis^30^. Here, we compared the PTX3 levels in the plasma among groups of colitis with and without adenosine, and found that PTX3 also increased significantly in the colitis and saline group while decreased in the adenosine treated rats with colitis, suggesting an appreciable effect of adenosine on the rectum with AA-induced acute colitis in rats.

## Conclusions

The findings of the present study reflect the high potential and remarkable therapeutic effects of adenosine as a new therapeutic strategy in inflammatory intestinal diseases. Adenosine signaling represents impactful levers to be tackled to improve the control of mucosal inflammation. Still, there are some issues that need to be examined, such as the clinical implication of adenosine in humans. Although this experimental study exerts substantial results, overall clinical studies for these novel concepts are still lacking. Therefore, further mechanism and molecular studies are needed to shed new light at the underlying mechanism of adenosine in the inflammatory diseases.

## Footnotes

1 Research performed at Experimental Medicine R & D Laboratory of Tubitak Gebze Campus, Gebze, Turkey.
